# Heat Shock Protein 90 Inhibitors Repress Latent Membrane Protein 1 (LMP1) Expression and Proliferation of Epstein-Barr Virus-Positive Natural Killer Cell Lymphoma

**DOI:** 10.1371/journal.pone.0063566

**Published:** 2013-05-03

**Authors:** Takayuki Murata, Seiko Iwata, Mohammed Nure Alam Siddiquey, Tetsuhiro Kanazawa, Fumi Goshima, Daisuke Kawashima, Hiroshi Kimura, Tatsuya Tsurumi

**Affiliations:** 1 Division of Virology, Aichi Cancer Center Research Institute, Nagoya, Aichi, Japan; 2 Department of Virology, Nagoya University Graduate School of Medicine, Nagoya, Aichi, Japan; The University of North Carolina at Chapel Hill, United States of America

## Abstract

Epstein-Barr virus (EBV) LMP1 is a major oncoprotein expressed in latent infection. It functions as a TNFR family member and constitutively activates cellular signals, such as NFκB, MAPK, JAK/STAT and AKT. We here screened small molecule inhibitors and isolated HSP90 inhibitors, Radicicol and 17-AAG, as candidates that suppress LMP1 expression and cell proliferation not only in EBV-positive SNK6 Natural Killer (NK) cell lymphoma cells, but also in B and T cells. Tumor formation in immuno-defficient NOD/Shi-scid/IL-2Rγ^null^ (NOG) mice was also retarded. These results suggest that HSP90 inhibitors can be alternative treatments for patients with EBV-positive malignancies.

## Introduction

The Epstein-Barr virus (EBV) is a human gamma-herpesvirus that mainly infects and establishes latent infection in B lymphocytes, but can also infect other types of cells, including NK, T and epithelial cells. EBV infection has been implicated as a causal factor in a variety of malignancies, and the expression pattern of viral latent genes varies depending on the tissue of origin and the state of the tumors. Neoplasms such as Burkitt's lymphomas or gastric carcinomas express only EBER and EBNA1 (type I latency), whereas some Hodgkin lymphomas, nasopharyngeal carcinomas (NPC) and NK/T lymphomas express EBER, EBNA1, LMP1 and LMP2 genes (type II latency). In addition to the type II genes, EBNA2, EBNA3 and EBNA-LP are also expressed in immunosuppression-related lymphomas or lymphoblastoid cell lines (LCLs) (type III latency).

EBV is associated with various types of T or NK cell lymphoproliferative diseases (T/NK LPDs). A severe form of chronic active EBV disease (CAEBV), mainly found in East Asia including Japan, is caused by clonal expansion of EBV-infected T or NK cells [1]–[3]. Others include extranodal NK/T lymphoma, nasal type (ENKL), and aggressive NK cell leukemia (ANKL). Although such EBV-positive T/NK LPDs are relatively rare, therapeutic treatment for those disorders is challenging, and the prognosis of those patients often can be dismal [4], [5]. Therefore, development of effective and specific drugs is an important goal.

The EBV latent infection integral membrane protein 1 (LMP1) is frequently expressed in latent EBV infections, including NK/T lymphomas. Since it functions as a constitutive TNFR family member by aggregation in the plasma membrane, resulting in constitutive activation of cellular signaling through NFκB, MAPK, JAK/STAT and AKT, LMP1 is assumed to be a major oncogene encoded by EBV [6]–[15].

Heat-shock protein 90 (HSP90) is an ATP-dependent molecular chaperone that is important for stability, quality control, protein interaction and functional maturation of cellular or viral client proteins. Because HSP90 is occasionally overexpressed and present in an activated form in cancer cells, and thereby supports proliferation of activated oncoproteins, including many cancer-associated kinases and transcription factors, it is regarded as an essential factor for oncogenic transformation [16], [17]. Radicicol and 17-AAG are HSP90 inhibitors which interact directly within its ATP-binding pocket, preventing ATP binding and interaction with client proteins [18]. These inhibitors might thus have potential as anti-cancer drugs for malignancies that depend on particular driver oncogene products that are sensitive HSP90 clients [16], [17], [19]. For example, HSP90 inhibitors have shown promise as anti-myeloma agents in pre-clinical settings and are currently being evaluated in clinical trials [20].

In the present study, we screened small molecule inhibitors and isolated HSP90 inhibitors as candidates that suppress LMP1 expression and cell proliferation in EBV-positive SNK6 NK cell lymphoma cells. The inhibitors not only retarded tumor proliferation at the culture level but also tumor formation in immuno-defficient NOD/Shi-scid/IL-2Rγ^null^ (NOG) mice. HSP90 inhibitors therefore may offer alternative treatments for EBV-positive malignancies.

## Materials and Methods

### Cell Culture and Reagents

An EBV-positive NK cell lymphoma line, SNK6, and an EBV-positive T cell line, SNT13, were maintained in RPMI1640 medium supplemented with 10% human serum (MP Biomedicals), 2 mM of Glutamax (GIBCO), 0.88 mM Oxalicacetic acid (SIGMA), 1 mM Sodium Pyruvate (GIBCO) and 700 U/ml of IL-2 (Primmune Inc.). SNK6 was originally established from a patient with ENKL and characterized by Nagata and others [21]. SNT13 is a γδ T-cell clone established from a patient with CAEBV [22]. These cell lines of low passage numbers were kindly provided by N. Shimizu in December 2009. B95-8 cells [23] were maintained in RPMI1640 medium supplemented with 10% fetal bovine serum. The Screening Committee of Anticancer Drugs (SCADS), Japan, kindly provided a library of small molecule inhibitors.

### Real-time PCR

For real-time RT-PCR, total cell RNA was purified using TriPure Isolation Reagent (Roche) and subjected to Real-Time RTPCR using a One Step SYBR PrimeScript RT-PCR Kit II (TaKaRa) with the Real-Time PCR System 7300 according to the manufacturer's instructions. PCR was performed as described earlier [24]. Primers used for the RT-PCR were as follows [25]: for GAPDH mRNA, 5′-TGCACCACCAACTGCTAGC-3′ and 5′-GGCATGGACTGTGGTCATGAG-3′; for LMP1 mRNA, 5′-CTATTCCTTTGCTCTCATGC-3′ and 5′-TGAGCAGGAGGGTGATCATC-3′; and for EBNA1 mRNA, 5′-AGGTACAGGACCTGGAAATG-3′ and 5′-CCTCGTCCATGGTTATCACC-3′. Real-Time PCR with GAPDH primers was also performed to serve as an internal control for input RNA.

### Antibodies and Immunoblotting

Anti-tubulin antibody was from Cell Signaling, and anti-LMP1 monoclonal antibody was reported previously [25]. For immunoblotting, cell proteins lysed in sample buffer were subjected to SDS-PAGE, followed by immunoblotting with the indicated antibodies as described previously [25].

### Transplantation of SNK6 into NOD/Shi-scid/IL-2Rγ^null^ (NOG) mice

Female NOG mice were purchased from the Central Institute for Experimental Animals, Japan. Twenty four NOG mice at the age of 10 weeks were subcutaneously implanted with 5×10^6^ SNK6 cells per mouse on day 0. From day 14, DMSO (vehicle) was injected into 12 mice, and the rest were treated with 17-AAG (6 times in two weeks, 50 mg/kg in total) intra-peritoneally. Peripheral blood was collected from the tail veins once in two weeks, and levels of EBV genomic DNA in whole blood were measured by Real-Time PCR as described previously [26]. Subcutaneous tumor masses were also measured with an external caliper and tumor volume was calculated using the formula: π × short axis × long axis × height/6. Animal experiments were approved by the University Committee in accordance with the Guidelines for Animal Experimentation at Nagoya University.

### Statistical analysis

Data shown are means ± standard errors and were analyzed using SPSS for Windows version 18.0 (IBM Corporation, Chicago, IL, USA). The therapeutic results were analyzed using the Mann-Whitney *U* test between groups.

## Results

### Identification of HSP90 inhibitors on screening for small molecule inhibitors that repress LMP1 transcription

**Figure 1:**
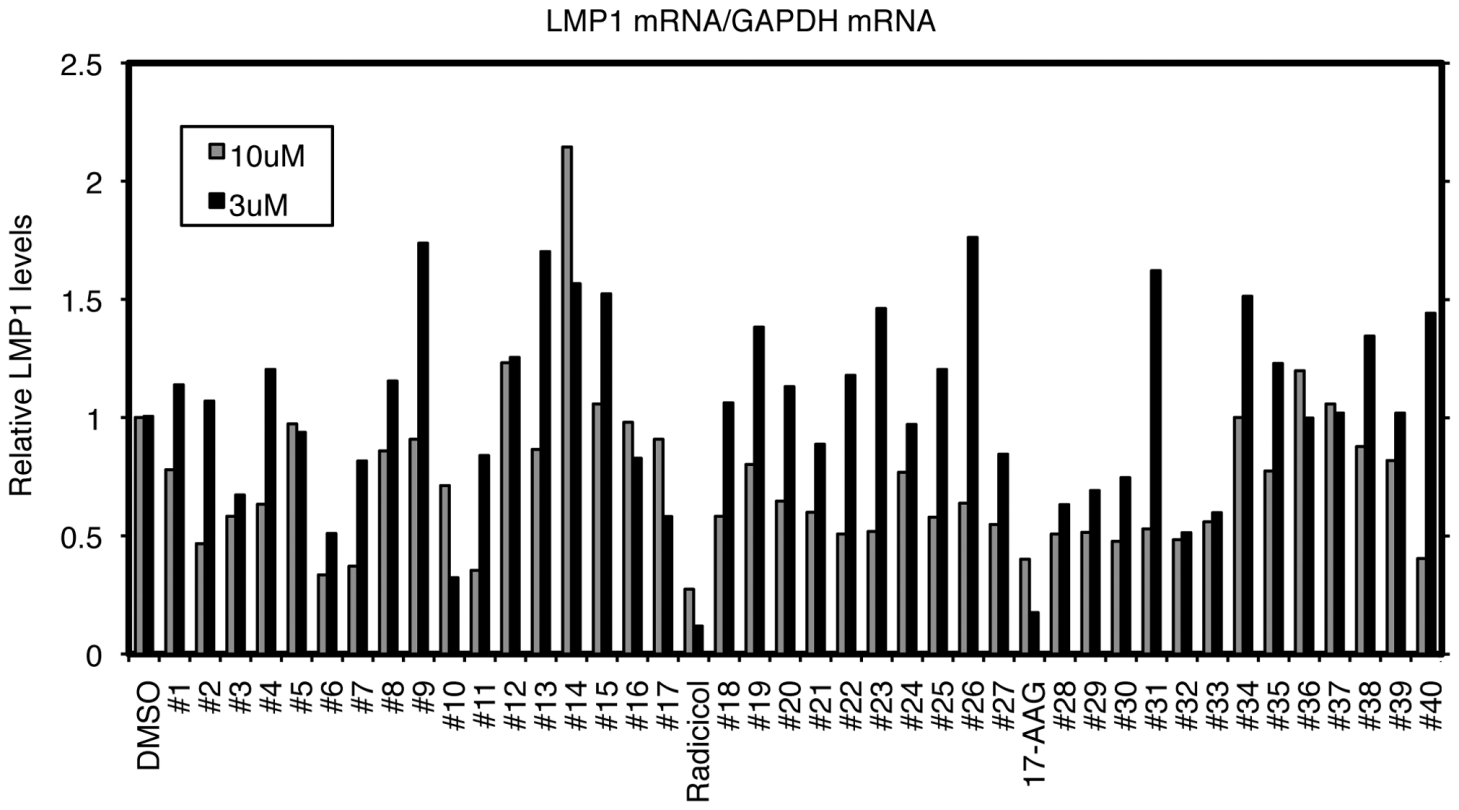
Representative example of screening for small molecule inhibitors that repress LMP1 expression. The EBV-positive NK lymphoma cell line, SNK6, was seeded and small molecule inhibitors were added to the media at concentrations of 10 and 3 µM. After 72 h, cell RNA was collected and subjected to Real-Time RT-PCR using specific primers for LMP1 and GAPDH mRNAs. Relative LMP1 mRNA levels are shown after normalization to GAPDH mRNA levels.

**Figure 2:**
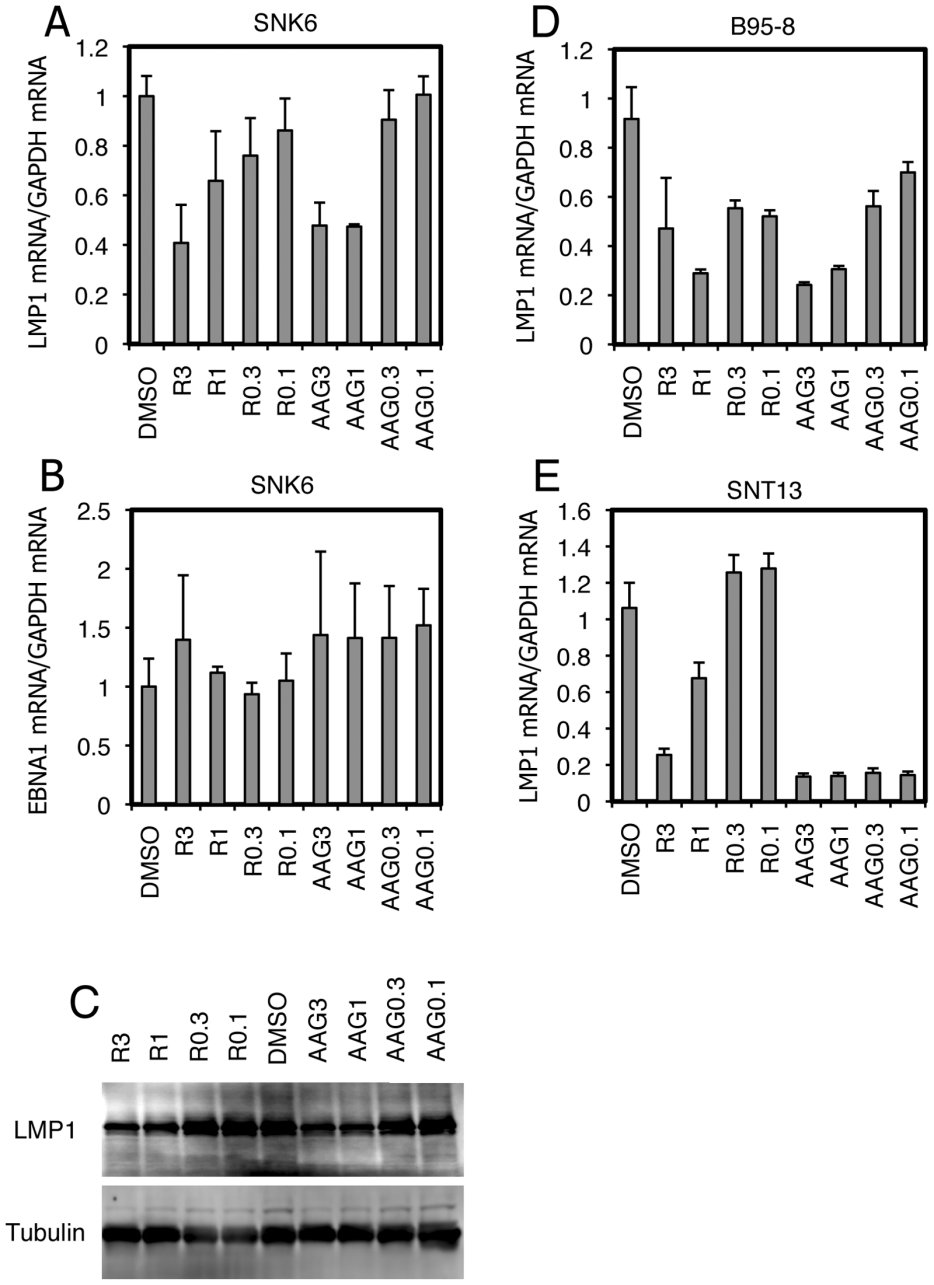
HSP90 inhibitors decrease LMP1 expression. (A, B) SNK6 cells were treated with Radicicol (R) or 17-AAG (AAG) at the concentrations of 3, 1, 0.3 or 0.1 µM. After 72 h, cell RNA was collected and subjected to Real-Time RT-PCR. Relative LMP1 levels (A) and EBNA1 levels (B) are shown after normalization to GAPDH mRNA levels. (C) SNK6 cells were treated with Radicicol (R) or 17-AAG (AAG) at the concentrations of 3, 1, 0.3 or 0.1 µM. After 72 h, cell proteins were collected and subjected to immunoblotting using anti-LMP1 and -Tubulin antibodies. (D, E) As in Fig. 2A, B95-8 (D) or SNT13 (E) cells were treated and subjected to Real-Time RT-PCR. Relative levels of LMP1 mRNA are shown after normalization to GAPDH mRNA levels. Each bar represents the mean and SD of three independent transfections.

CAEBV and ENKL committed to type II latency express the major EBV oncogene, LMP1. We therefore conducted a search for small molecular inhibitors that repress expression of LMP1. As an initial screen, the EBV-positive NK cell lymphoma, SNK6, was treated with chemicals or the vehicle DMSO at concentrations of 3 or 10 µM for 3 days. Cellular RNAs were harvested and subjected to Real-Time RT-PCR. Among 300 small molecule substances with identified targets, we found Radicicol and 17-AAG, inhibitors of HSP90, to decrease LMP1 transcripts (Fig. 1). In order to further evaluate the effect, SNK6 cells were administered 3, 1, 0.3, 0.1 µM of Radicicol or 17-AAG (Fig. 2A–C). LMP1 levels decreased to 41% and 48% of control (DMSO) with 3 µM of Radicicol and 17-AAG, respectively (Fig. 2A), with EBNA1 levels appearing relatively unaffected (Fig. 2B). We then examined protein levels of LMP1 in Fig. 2C. After 72 h with 3 or 1 µM of Radicicol or 17-AAG, LMP1 protein levels were decreased in SNK6 cells (Fig. 2C), in a similar fashion with the transcript levels. LMP1 expression in B95-8 or EBV-positive T cell line (SNT13) was also reduced by these HSP90 inhibitors (Fig. 2D, E). For unknown reasons, 17-AAG markedly suppressed LMP1 expression from SNT13 cells even at lower concentrations (Fig. 2E).

### Suppression of cell proliferation *in vitro* and *in vivo*

**Figure 3:**
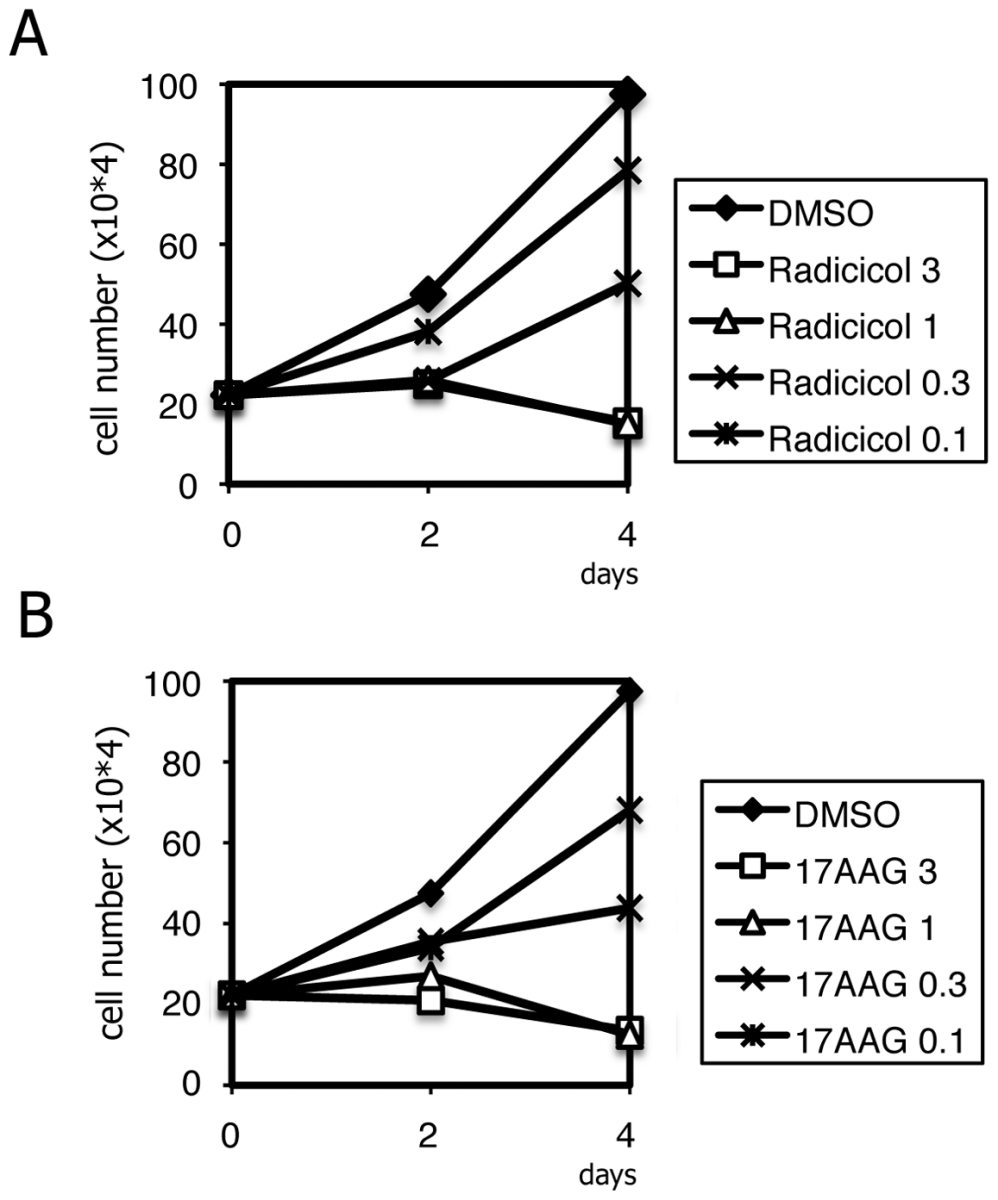
HSP90 inhibitors suppress cell proliferation of the EBV-positive SNK6 NK lymphoma line. SNK6 cells were cultured with Radicicol (A) or 17-AAG (B) at concentrations of 3, 1, 0.3 or 0.1 µM and cell numbers were counted on days 0, 2 and 4. Data are shown as the means of three independent replicates.

After confirming that HSP90 inhibitors repress LMP1 expression in EBV-positive lymphoma cells, we then examined whether the inhibitors might actually suppress cancerous growth. We first tested at the cell culture level (Fig. 3). Three or 1 µM of Radicicol (Fig. 3A) or 17-AAG (Fig. 3B) could fully block proliferation of SNK6 cells depending on the concentration. Partial but appreciable inhibition was also exhibited at lower concentrations.

**Figure 4:**
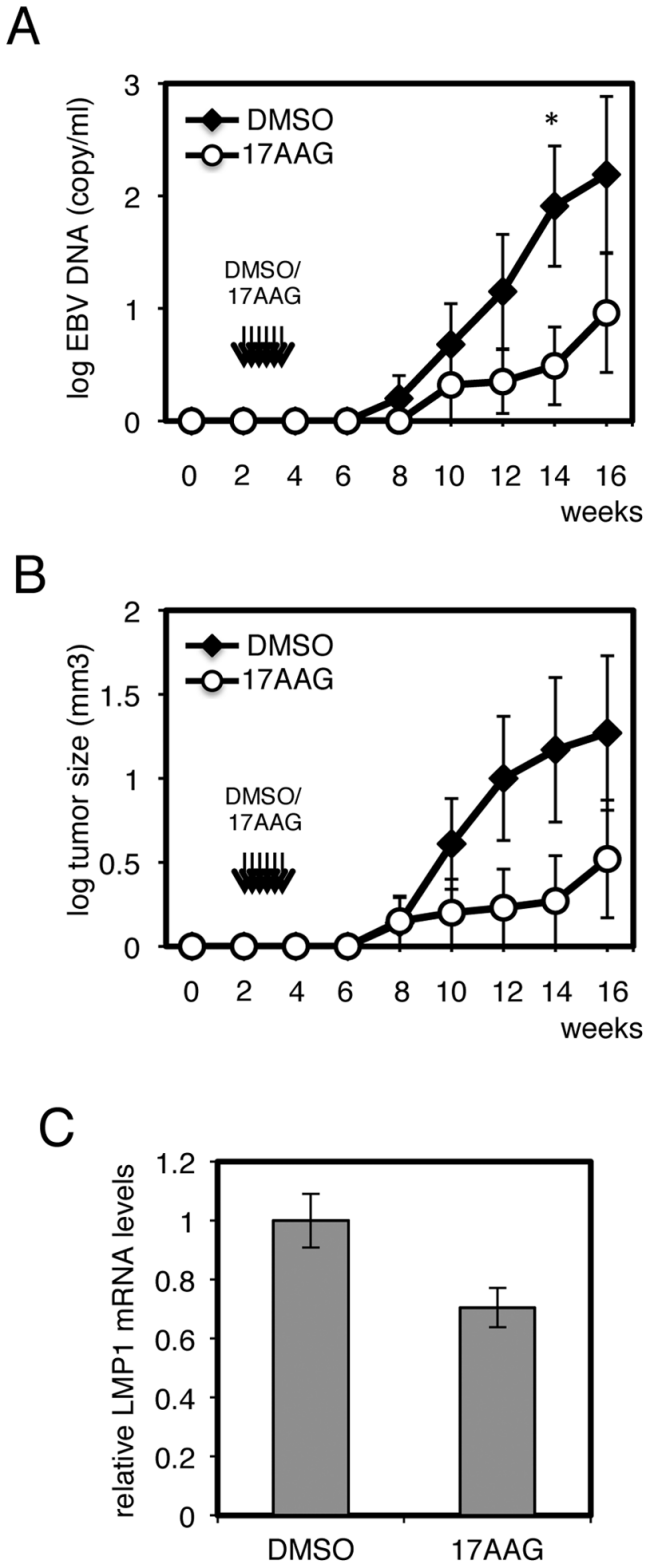
Effects of HSP90 inhibitors *in vivo*. HSP90 inhibitors repress EBV copy numbers in the whole blood (A) and tumor size (B) in mice implanted with EBV-positive NK cell lymphoma. Twenty-four NOG mice were subcutaneously implanted with 5×10^6^ of SNK6 cells per mouse on day 0. From day 14, DMSO (vehicle) or 17-AAG (6 times in two weeks, 50 mg/kg in total) was injected to twelve mice each, intra-peritoneally. Peripheral blood was collected from the tail veins once in two weeks, and levels of EBV genomic DNA in whole blood were measured by Real-Time PCR (A). Subcutaneous tumor masses (B) and LMP1 mRNA levels in the tumors (C) were also measured. *p<0.05 (Mann-Whitney *U* test).

It has been already reported that HSP90 inhibitors block proliferation of EBV-positive malignancies, including NK/T lymphomas [27], [28], although the earlier studies did not test LMP1 levels. For examination of effects *in vivo*, we here adopted a novel mouse xenograft model using severely immuno-deficient mice of the NOG strain [29], allowing assessment of whether the HSP90 inhibitors might repress EBV-positive malignant cells without seriously affecting other normal part of tissues and organs. The NOG mice were injected subcutaneously with 5×10^6^ of SNK6 cells at day 0, and low doses of 17-AAG (50 mg/kg in total) or DMSO were administered into the abdominal cavity for 6 times between days 14 and 25. EBV genomic DNA titers in whole blood (Fig. 4A) and tumor sizes (Fig. 4B) were measured. As shown in Fig. 4, 17-AAG markedly reduced the viral titer in blood and growth of the NK lymphoma cells in NOG mice. Because engraftment of NK lymphomas in mice is not very efficient [29], only 50% of the mice developed subcutaneous tumor masses and others failed to nurture the lymphoma cells even in the DMSO treatment group. Due to this low efficiency of transplantation, statistical significance could be exhibited only once for EBV DNA load (Fig. 4A, 14 weeks), but we gained the clear impression that 17-AAG worked more efficiently. This is an important difference when compared to LCLs, with which 100% of injected mice develop tumors, and thereby the efficacy of 17-AAG was exhibited more significantly [28]. We then compared the LMP1 expression levels in the tumor developed in the absence or presence of 17-AAG (Fig. 4C). LMP1 transcript was lower in the tumor treated with 17-AAG, indicating that the inhibitor reduced LMP1 transctiption *in viv*o, too. To summarize, our results imply high potential of HSP90 inhibitors for treating EBV-positive cancers, including EBV-positive NK lymphomas.

## Discussion

ENKL is an aggressive type of cancer often associated with resistance to chemotherapy, and accordingly a poor prognosis. Therefore, treatments are needed that specifically target its molecular determinants. We here focused on expression of the major oncogene of EBV, LMP1, and found two HSP90 inhibitors, Radicicol and 17-AAG, to both decrease LMP1 expression and cancerous growth of the EBV-positive SNK6 NK cell lymphoma line, *in vitro* and *in vivo*. Although the reduction in LMP1 levels by the inhibitors correlated with cell growth inhibition, we still cannot tell if low levels of LMP1 is responsible for the growth inhibition.

Jeon et al. previously reported that HSP90 inhibitors induce apoptotic cell death in EBV-positive NK/T lymphoma cells in vitro [27], but did not examine effects *in vivo*. Elsewhere, Sun et al. reported that HSP90 inhibitors block proliferation *in vitro* and *in vivo* mostly using EBV-positive LCLs [28], but NK/T lymphomas were not tested. Importantly, unlike ours, both of the previous papers did not include data on LMP1 levels.

**Figure 5:**
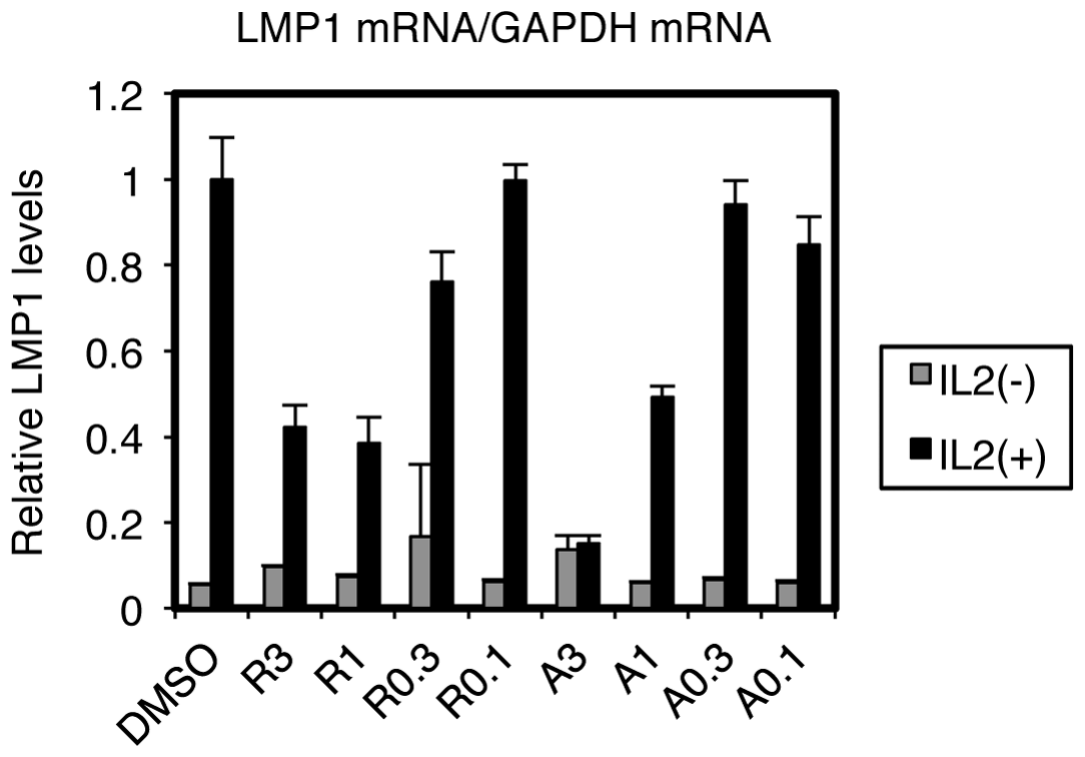
Suppression of LMP1 expression in EBV-positive NK cells by HSP90 inhibitors is likely dependent on IL-2. SNK6 cells, routinely cultured with IL-2, were washed extensively with PBS, and then cultured in the presence (black bars) or absence (gray bars) of IL-2 with Radicicol (R) or 17-AAG (A) at concentrations of 3, 1, 0.3 or 0.1 µM. After 72 h, cell RNAs were collected and subjected to Real-Time RT-PCR. Relative levels of LMP1 mRNA are shown after normalization to GAPDH mRNA levels. Each bar represents the mean and SD of three independent transfections.

LMP1 gene transcription differs between type II and type III latency infection. In the latter, LMP1 transcription is turned on by EBNA2 [30]–[32], whereas LMP1 expression is independent of EBNA2 in type II. In the previous work of Jeon and others induction of apoptosis by HSP90 inhibitors was considered to be through AKT signaling inhibition [27]. The decreased expression of LMP1 gene we observed here (Fig. 1, 2) might have been brought about through suppression of the AKT pathway. In latency II, including the ENKL case, it has been frequently reported that cytokines, such as IL-4, IL-6, IL-10, IL-13 and IL-21, activate the JAK/STAT pathway, thereby inducing LMP1 gene expression through STAT [33]–[38]. Because JAK/STAT signaling can also be blocked by HSP90 inhibitors [39], it may also be involved. Actually, since our EBV-positive NK/T cells are cultured routinely with IL-2 and it is involved in LMP1 expression [40], the cytokine and its downstream signaling may also be implicated in the reduction by HSP90 inhibitors. To examine this, we cultured SNK6 cells with or without IL-2 and HSP90 inhibitors, and measured the levels of LMP1 mRNA (Fig. 5). As expected from the previous report [40], depletion of IL-2 down-regulated LMP1 levels to 5.5% of control, in DMSO-treated cells. Treatment with Radicicol (R) or 17-AAG (A) at 1 µM or higher caused reduction of LMP1 levels in the presence of IL-2 (black bars), but it did not significantly decrease the levels without IL-2 (gray bars). This result suggests that HSP90 inhibitors suppress LMP1 expression, which is activated by IL-2, and that the cell signalings elicited by IL-2, such as JAK/STAT, are likely be responsible for the LMP1 reduction by HSP90 inhibitors. Besides JAK/STAT pathways, NFκB signaling must also be notified, because it is known that IL-2 elicits NFκB signaling [41], and HSP90 inhibitors can repress NFκB [42], [43]. In addition, C/EBP contributes to LMP1 expression in type II [25], and may also be regulated by HSP90 inhibitors.

In type III latency, although EBNA2 does not feature DNA binding activity, it enhances LMP1 promoter activity by acting as a cofactor. It associates with cellular transcriptional factors, including the Recombination signal Binding Protein Jκ (RBP-Jκ [32], [44] and PU-box 1 (PU.1) (also known as Spleen Forming Virus (SFV) Proviral Integration 1 (SPI1)) [30], [31], [45], [46], which are then recruited onto the LMP1 promoter for transactivation. Because we found LMP1 expression was decreased by HSP90 inhibitors even in type III B95-8 cells (Fig. 2B), RBP-Jκ or PU.1 may also be under the control of HSP90. In fact, PU.1 is known to be inhibited by HSP90 inhibitors [47], [48].

In summary, we here observed reduced expression of LMP1 and simultaneous growth suppressive effects of Radicicol and 17-AAG in EBV-positive lymphomas, especially NK lymphoma. Although the molecular mechanisms of how the HSP90 inhibitors block cell proliferation are elusive because HSP90 has a number of client proteins, our observation suggests that they could be potent therapeutic drugs for EBV-positive lymphomas. Rapid progress in the field of Hsp90 biology has brought about development of more potent and less toxic inhibitors. It is to be hoped that these may become useful as antiviral drugs against EBV-associated disorders.
